# The Diversity and Biogeography of Western Indian Ocean Reef-Building Corals

**DOI:** 10.1371/journal.pone.0045013

**Published:** 2012-09-19

**Authors:** David Obura

**Affiliations:** CORDIO East Africa, Mombasa, Kenya; University of Texas, United States of America

## Abstract

This study assesses the biogeographic classification of the Western Indian Ocean (WIO) on the basis of the species diversity and distribution of reef-building corals. Twenty one locations were sampled between 2002 and 2011. Presence/absence of scleractinian corals was noted on SCUBA, with the aid of underwater digital photographs and reference publications for species identification. Sampling effort varied from 7 to 37 samples per location, with 15 to 45 minutes per dive allocated to species observations, depending on the logistics on each trip. Species presence/absence was analyzed using the Bray-Curtis similarity coefficient, followed by cluster analysis and multi-dimensional scaling. Total (asymptotic) species number per location was estimated using the Michaelis-Menten equation. Three hundred and sixty nine coral species were named with stable identifications and used for analysis. At the location level, estimated maximum species richness ranged from 297 (Nacala, Mozambique) to 174 (Farquhar, Seychelles). Locations in the northern Mozambique Channel had the highest diversity and similarity, forming a core region defined by its unique oceanography of variable meso-scale eddies that confer high connectivity within this region. A distinction between mainland and island fauna was not found; instead, diversity decreased radially from the northern Mozambique Channel. The Chagos archipelago was closely related to the northern Mozambique Channel region, and analysis of hard coral data in the IUCN Red List found Chagos to be more closely related to the WIO than to the Maldives, India and Sri Lanka. Diversity patterns were consistent with primary oceanographic drivers in the WIO, reflecting inflow of the South Equatorial Current, maintenance of high diversity in the northern Mozambique Channel, and export from this central region to the north and south, and to the Seychelles and Mascarene islands.

## Introduction

The reef-building coral fauna of the Western Indian Ocean (WIO) is one of the least known globally. Dedicated taxonomic and diversity studies are spread over a broad period of time and have tended to be geographically constrained, such as for Tanzania [1], South Africa [2], [3], southwest Madagascar [4], Seychelles [5] and the Mascarene Islands [6]–[8]. A greater number of studies have recently been undertaken in the northern parts of the Indian Ocean, including the Red Sea and Gulfs regions e.g. [9]–[11], Yemen [12], [13], Socotra [14], Oman [15], the Lakshadweep archipelago [16] and the Maldives [17]. Using datasets from studies with highly unequal levels of sampling, the main regional coral distribution analyses [18], [19] found that coral species diversity across the Indo-Pacific region is one of approximately linear decline in all directions from the high-diversity center in the southeast Asian region, currently known as the Coral Triangle [20]. Sheppard [18] noted that this decline does not hold for the Red Sea, finding a regional peak in diversity in the Red Sea, but species richness for mainland East Africa and adjacent island sites showed a clear decline. Recently work in the WIO and Red Sea has highlighted a number of new records for the region as well as previously unknown coral species [21]–[23], and cryptic species or genetic disjunctions between Indian and Pacific Ocean populations [24]–[27].

**Figure 1:**
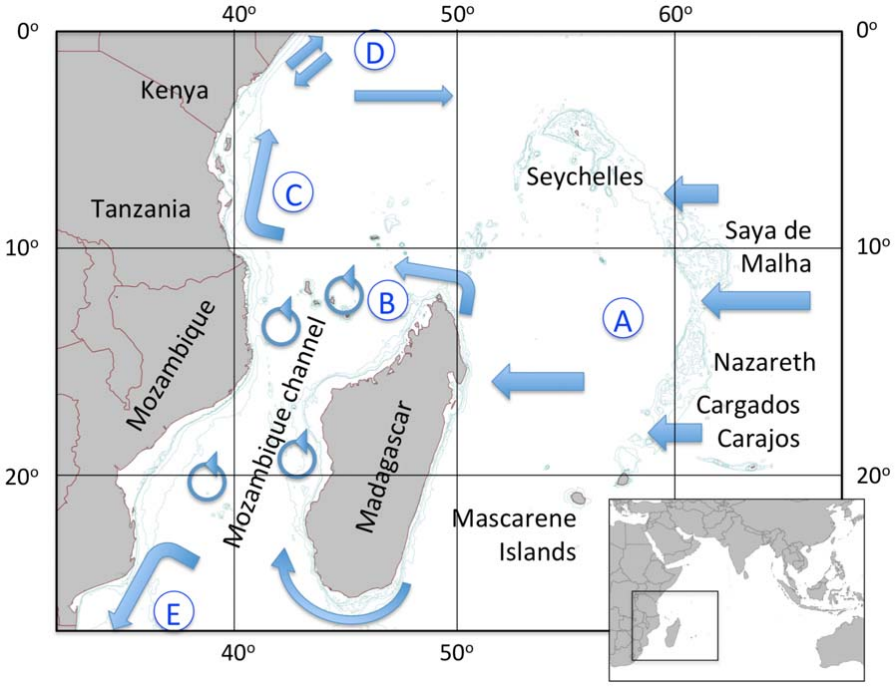
The Western Indian Ocean, defined by the East African coast and the Saya de Malha, Nazareth and Cargados Carajos banks of the Mascarene Plateau. The principal currents that define the region are coded by the circled letters A–E (see Table 1). Bathymetric contours were selected to illustrate the main plateau and bank features, at 60, 200 and 1000 m depth.

Biogeographic patterns are determined by past and present day currents that define connectivity among sites, as well as by historical patterns of speciation, extinction and immigration. The present-day oceanography of the WIO [28] can be characterized as follows (fig. 1, Table 1). The east-west flow of the South Equatorial Current (SEC) carries waters from the Indonesian region across the Indian Ocean between 10–20°S, with primary flow centered at 12–13°S through the gap between the Saya de Malha and Nazareth banks [29] and at about 16–17°S where it reaches the Madagascan coastline and bifurcates north and south. The zonal flow of the SEC isolates the northern (Seychelles) and southern (Mascarene islands) islands and banks from the main flow and from each other, and establishes a clear upstream-downstream gradient between these and the Mozambique Channel and East Africa mainland coast. On the east coast of Madagascar the northern branch of the SEC accelerates around the northern tip of Madagascar, where instabilities in the current result in the formation of the Glorioso Front [30], the intermittent Comoros gyre [31] and mesoscale eddies [32] that result in high mixing of waters in the northern Mozambique Channel. Water flows out from the northern Mozambique Channel in both northerly and southerly directions - northwards in the linear East African Coastal Current (EACC) that flows along the Tanzania and Kenya coasts, and southwards as a net flow of the variable eddies initiated in the northern Mozambique Channel. These eddies subsequently join up with waters from the East Madagascar Current to form the Agulhas Current that flows south into the temperate belt on the South African coast [33]. In the north the reversal of monsoon winds and the Somali Current result in complex interactions with the EACC, which from May to October continues northwards to join the gyre that circulates in the northern Indian Ocean. From December to April the EACC turns east at about 2°S forming the Equatorial Counter Current that flows towards the northern Seychelles and Maldives/Lakshadweep archipelagos. These result in mixing of WIO waters with those from the Red Sea, Gulf of Aden and Arabian and Omani gulfs and West Indian regions. As a result of upwelling in the northern Indian Ocean, the transition from the warm EACC to the cool waters of this region mirrors the tropical to temperate transition from the Mozambique Channel to the Agulhas current, in the south.

**Table 1:** Principle currents of the Western Indian Ocean, their relevance and consequences for the biogeography of the reef building coral fauna.

Main current	Biogeographic relevance	Consequence	Evidence
A) South Equatorial Current (SEC)	Immigration of species/gene flow from the Central Indo-Pacific Realm, similarity of Chagos/northern Mozambique Channel fauna, species accumulation in the WIO.	High diversity in WIO	[18], [19] this study
	Isolation and low diversity of islands north (Seychelles) and south (Mascarene islands) of the SEC.	Low diversity and endemism in the islands	[41], [42] this study
B) Eddies and the Comoros Gyre in the Mozambique Channel	High connectivity within the northern Mozambique Channel (NMC); high retention of larvae and genetic diversity within the channel.	High species richness in the northern Mozambique Channel	[22], [96] this study
	High connectivity within the southern Mozambique Channel, higher productivity due to eddy-shelf interactions, mixing with the East Madagascar Current and upwelling from the South Madagascar Plateau	Transition to colder/higher nutrient fauna and habitats. Lower coral diversity	[44], [45]
C) East Africa Coastal Current (EACC)	Linear transport from NMC to 2°S (north Kenya) then monsoon current reversals and influence of Somali upwelling system.	Declining diversity with distance, transition to different coral fauna	[1], [46] this study
D) Somali Current, monsoon reversals &upwelling	Monsoon current reversals, Somali upwelling system, exchange with the northern Indian Ocean (Red Sea, Gulfs of Aden/Oman/Arabia)	Different coral fauna, colder/higher nutrient conditions and extreme environments.	[11], [14], [15], [97]
E) Agulhas Current	Merging of Mozambique Channel waters and East Madagascar Current to form the Agulhas Current.	Colder/marginal conditions, declining coral diversity	[97], [98]

**Figure 2:**
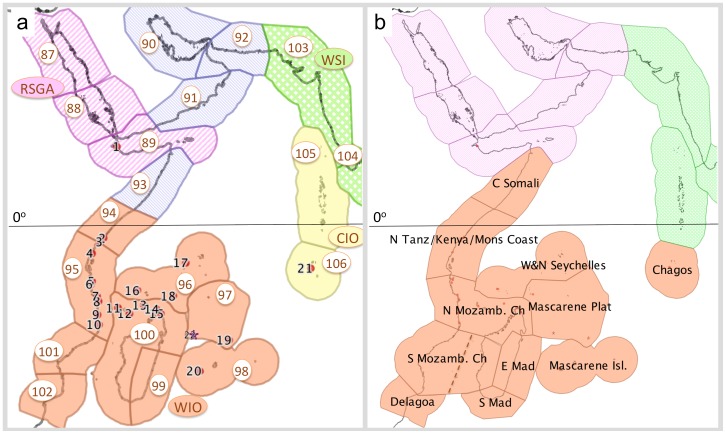
Ecoregions and provinces of the Western Indo-Pacific Realm, excluding the Andaman Seas in the East. a) Marine Ecoregions of the World (MEOW) [36] provinces and ecoregions (abbreviations and numbers in circles), and sample locations (numbers, see Table 2). MEOW province and ecoregion codes (see Table 2 and fig. 4): *Province - Red Sea/Gulf of Aden (RSGA*): ecoregions - North Central Red Sea (87), Southern Red Sea (88), Gulf of Aden (89, GA); *Province - Somali-Arabian Seas (SAS)*: ecoregions - Persian (90), Oman (91), West Arabian Sea (92), Central Somali (93); *Province - Western Indian Ocean (WIO)*: ecoregions - North Monsoon Current (94, Mons), East African Coral Coast (95, EAC), Seychelles (96, Sey), Cargados/Tromelin (97, Car), Mascarene Islands (98, Mas), Southeast Madagascar (99), West & North Madagascar (100, W&NM), Sofala (101), Delagoa (102); *Province - West & South India (WSI)*: ecoregions - West India (103), South India/Sri Lanka (104); *Province - Central Indian Ocean (CIO)*: ecoregions - Maldives (105), Chagos (106). The star shows the position of Tromelin Island (France). b) adjustments to the MEOW ecoregions and provinces suggested by this study (see Table 4) and discussion. A possible east-west split in the southern Mozambique Channel is shown by a dotted line.

Sources of evidence for the biogeographic patterns are listed in the table, and discussed in the text. See main text for references relevant to oceanographic features. Letter codes for each current correspond to letters in fig. 2a.

Understanding biogeographic patterns and their causal mechanisms provides the foundation for conservation planning. Kelleher et al. [34] laid the basis for recent marine biogeographic analyses, identifying East Africa as a coherent region (here referred to as the Western Indian Ocean). While that study joined the East African coast and Madagascar as a single region, further work based on practical planning for transboundary conservation separated the mainland [35], from the oceanic islands and Madagascar (RAMP-COI, unpublished). This work was most recently revised in the Marine Ecoregions of the World (MEOW) classification [36], which established a global hierarchy of 12 realms, 62 provinces and 232 ecoregions, and defines the WIO province as used in this study, extending from central Somalia to northern South Africa on the East African coast (including Kenya, Tanzania and Mozambique), and extending east as far as the banks of the Mascarene Plateau (Saya de Malha, Nazareth and Cargados Carajos) and the Mascarene Islands (fig. 1), and incorporating 9 ecoregions (fig. 2a). This classification is used for marine species in the IUCN Red List of Endangered species [37], which has compiled the most recent and publicly available biogeographic dataset of scleractinian corals as part of its assessment of coral species extinction risk [38]. Nevertheless, the MEOW ecoregions are at a relatively coarse scale for local considerations of biogeography and conservation planning. To address this finer scale, a dataset on coral species presence/absence compiled over a decade of surveys at locations spread throughout the WIO is presented here. This study therefore builds on the MEOW classification [36] through analysis first of the global IUCN dataset on coral species [38], then of the field dataset on coral distributions reported here, in the light of principal oceanographic drivers of biogeographic pattern (Table 1). As a result of this analysis, revisions to currently accepted biogeographic patterns are suggested.

## Results

### Red List coral distributions

**Figure 3:**
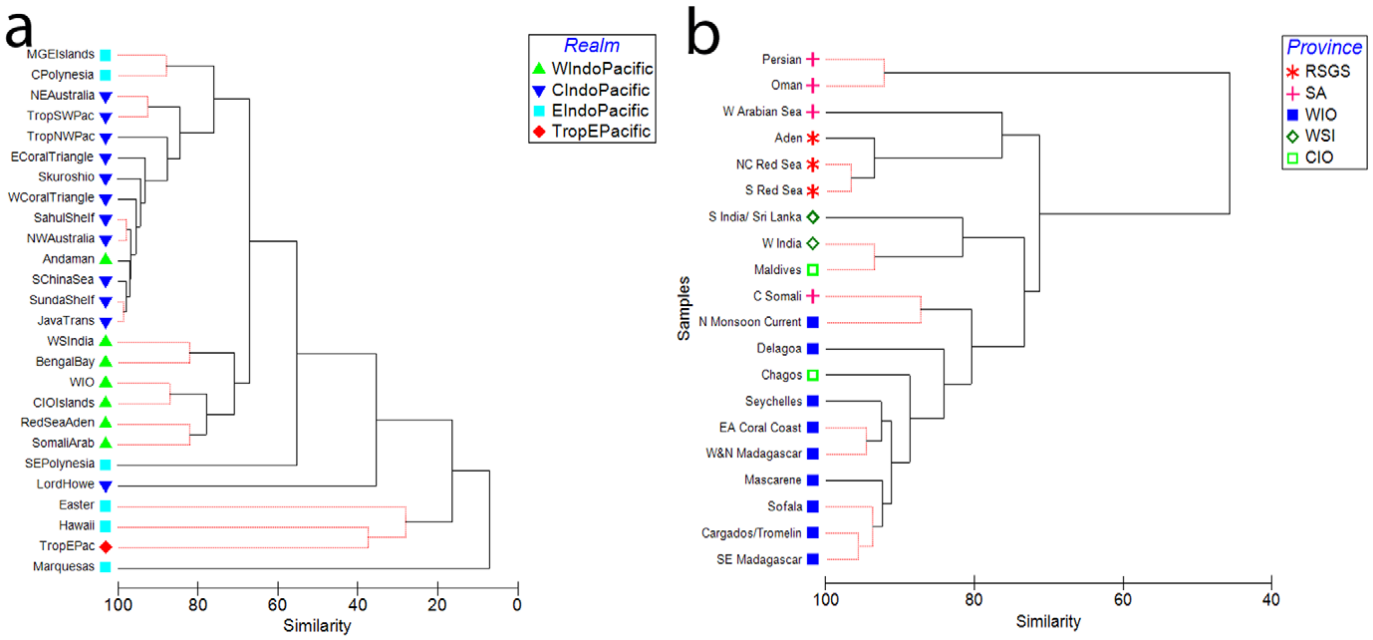
Cluster analysis of coral species from the IUCN Red List of Threatened species by MEOW provinces and ecoregions. a) All provinces in the tropical Indo-Pacific coded by Realm, and b) ecoregions within the West Indo-Pacific Realm, excluding the Andaman Seas, coded by province (see caption to fig. 2 for full province names). Significant groupings shown as thin red lines: a) ANOSIM, R = 0.609, p<0.001; b) ANOSIM R = 0.917, p<0.01; Primer v 6.0.

Across all tropical MEOW provinces, the Red List dataset on corals shows a significant segregation of provinces between the four Indo-Pacific Realms (ANOSIM, R = 0.558, p<0.001). Provinces within the West Indo-Pacific realm (WIPR) cluster strongly together (fig. 3a), except for the Andaman Seas (Andaman and Nicobar Islands and Western Sumatra). When this province is classified in the Central Indo-Pacific realm, a more significant result is obtained (ANOSIM, R = 0.609, p<0.001), so for subsequent analyses, the Andaman Seas ecoregions were excluded. For ecoregions within the WIPR six significant clusters were found (fig. 3b, ANOSIM R = 0.917, p<0.001), though these only group pairs of locations, and in one case 3 locations. Running an ANOSIM grouping ecoregions by the four MEOW provinces within the WIPR (excluding the Andaman Seas) gave a significant result (ANOSIM R = 0.787, p<0.01), and a comparable R statistic was obtained when ecoregions were grouped into three clusters suggested by the dendrogram (fig. 3b) (ANOSIM R = 0.788, p<0.01) as follows: a) the WIO ecoregions plus Chagos and the Central Somali coast, b) Maldives and the West and South India ecoregions, and c) the remaining ecoregions from the Red Sea, Gulfs of Aden and Somali-Arabian Seas provinces.

### Field data on coral distributions

Following reconciliation of field identifications across 21 study locations (Table 2), 413 distinct species or colony morphologies were noted, including a number of unidentified records that could not be assigned a species name either in situ or in post-processing of photographs and records. Excluding these uncertain records resulted in 369 stable identifications, including working identifications recorded as numbered records (e.g. ‘sp.1’) that were used consistently across locations.

**Table 2:** Survey locations in the Western Indian Ocean.

Country	Location	#	MEOW ecoregion	Year sampled	Number of samples	Species richness			Coordinates	
						Sampled	Smax	B	Longitude (E)	Latitude (S)
Comoros	Comoros	11	W&NM	2011	15	195	223	2.18	43.39	11.78
Djibouti	Djibouti	1	GA	2010	23	177	194	2.98	43.33	11.50
France	Glorieuse	13	W&NM	2011	9	159	209	2.88	47.25	11.55
	Mayotte	12	W&NM	2005/10	36	249	274	4.35	45.12	12.78
	Tromelin	2	Carg	2011	2	33	n/a	n/a	54.51	15.90
	Reunion	20	Masc	2011	9	123	205	5.89	55.40	21.10
Kenya	KenyaS	4	EAC	2001–6	14	203	239	2.9	39.73	4.02
	Kiunga	2	Mons	2002/5	37	167	177	3.51	41.40	1.80
	Lamu	3	Mons	2003/5	17	176	203	3.12	40.92	2.36
Madagascar	Diego-Vohemar	15	W&NM	2010	36	269	293	3.31	49.72	12.76
	Nosy Hara	14	W&NM	2008	16	212	230	1.76	49.07	12.13
Mauritius	St. Brandon	19	Carg	2010	17	157	185	3.58	59.63	16.61
Mozambique	Nacala	10	EAC	2011	12	220	297	4.08	40.66	14.25
	Pemba	9	EAC	2003/11	15	254	288	2.14	40.69	11.12
	Vamizi	8	EAC	2011	7	207	269	2.02	40.66	11.00
Seychelles	Aldabra	16	Sey	2002/8	20	190	215	2.97	46.28	9.40
	Amirantes	17	Sey	2009	28	190	217	4.18	53.33	5.41
	Farquhar	18	Sey	2009	28	139	174	7.31	51.50	10.20
Tanzania	Mafia	5	EAC	2004/7	27	265	280	2.12	39.75	8.10
	Mnazi	7	EAC	2003/9	32	265	276	2.46	40.45	10.27
	Songo	6	EAC	2003/9	29	235	250	2.36	39.60	8.50
UK	Chagos	21	Cha	2006	27	217	228	2.03	71.50	6.20

Survey details are shown for the reef-building coral dataset presented in this study, (columns from left to right): country, location and number (see fig. 2a), MEOW ecoregion (see fig. 2 caption for ecoregion codes [36]), year(s) of sampling, number of dive samples (see methods for details), coral species richness and geographic coordinates. Detailed results are shown for species richness, including the number of species sampled, and coefficients of the Michaelis-Menten equation based on the species accumulation curves: Smax = asymptotic number of species (expected maximum richness), and B = number of locations at which Smax/2 is predicted.

**Figure 4:**
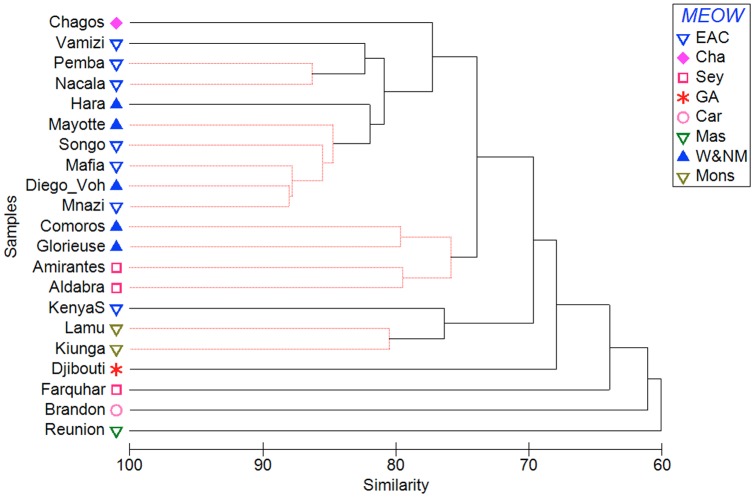
Cluster analysis of coral species presence/absence for study locations in the Western Indian Ocean. Study locations are listed in Table 2 and coded by MEOW ecoregion. Significant clusters are joined by thin red lines (ANOSIM R = 0.837, p<0.001).

The locations with the most similar coral faunas were Mafia Island, the Songosongo islands and Mnazi Bay (Tanzania), Diego-Vohemar (northeast Madagascar) and Mayotte (Comoro archipelago) forming the inner-most significant cluster (84.7% similarity, fig. 4). Adjacent to these is Nosy Hara (northwest Madagascar), a significant cluster comprising Pemba and Nacala, and Vamizi (all in northern Mozambique), at 82.3% similarity. Geographically, these define a northern Mozambique Channel region with an extension north to Mafia Island. This cluster groups together with Chagos at 77.2% similarity. A third significant cluster is formed by the Comoro islands (Grande Comore and Moheli) and Glorieuse island in the northern Mozambique Channel and the Aldabra and Amirantes groups of islands in western Seychelles, at 75.8% similarity. This islands cluster is most closely related to the northern Mozambique Channel cluster at 73.4% similarity, the two clusters overlapping geographically in the Comoros and Glorieuse. Beyond this main cluster of locations is a loose cluster comprising Lamu and Kiunga in northern Kenya (in a significant two-site cluster) with southern Kenya, then progressively dissimilar outliers: Djibouti, Farquhar atoll (southern Seychelles), St. Brandons island (Mauritius, on the Cargados Carajos bank) and Reunion island.

The dendrogram (fig. 4) does not match classification of locations by their MEOW ecoregion in three main ways; a) West and North Madagascar and East Africa Coral Coast locations are fully mixed at the highest level of similarity in the northern Mozambique Channel (>82% similarity), with close links to the Chagos ecoregion and the nearby Seychelles ecoregion (Aldabra and Amirantes groups, >75% similarity), b) the Northern Monsoon Current sites in northern Kenya are grouped with the south coast of Kenya on the East African Coral Coast at 76.3% similarity and then with the Gulf of Aden; and c) the isolated islands St. Brandons, Farquhar and Reunion, in the Cargados/Tromelin, Seychelles and the Mascarene ecoregions respectively, are outliers to these. The grouping of locations determined by the cluster analysis (ANOSIM R = 0.837, p<0.001) was more significant than when locations were grouped by their MEOW ecoregion (R = 0.709, p<0.001).

**Figure 5:**
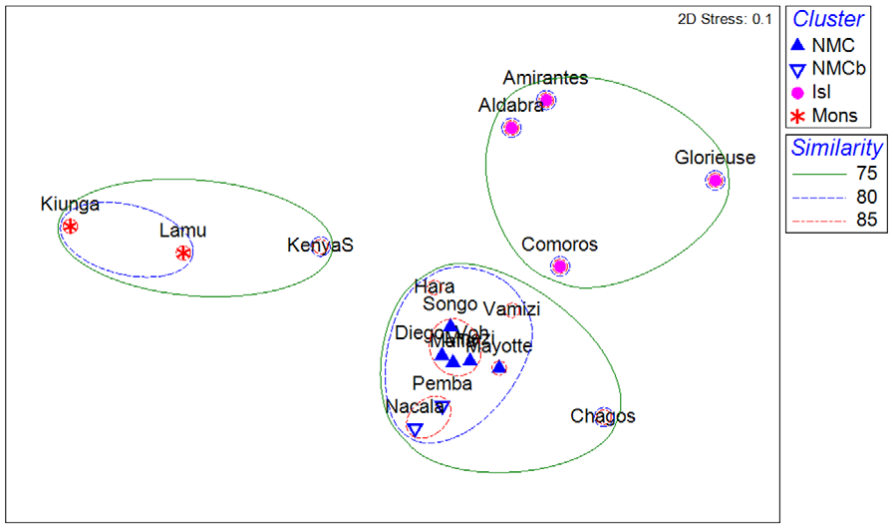
Multi-dimensional scaling (MDS) bi-plot of locations in the Western Indian Ocean excluding the outliers from Djibouti to Reunion island (from **fig. 4**). The outliers were excluded to more clearly represent relationships among the more highly related locations. Significant groups of locations (ANOSIM R = 0.837, p<0.001) coded as Northern Mozambique Channel (NMC), Northern Mozambique Channel group b (NMCb), islands in the northern Mozambique Channel and western Seychelles (Isl) and Northern Monsoon current (Mons). Similarity contours are shown at 75, 80 and 85% similarity.

The locations in the main cluster of fig. 4 (excluding outliers) are further illustrated using MDS (fig. 5). The main northern Mozambique Channel cluster (NMC) stands out clearly, with the closely related ‘b’ group of Pemba and Nacala (NMCb) within the 80% similarity contour. The islands in the northern Mozambique Channel and adjacent island groups in the Seychelles (Isl) are also clearly distinguished, at 75% similarity. The significant grouping of Kiunga and Lamu (Northern Monsoon coast, Mons) is grouped with southern Kenya, which is placed in the MDS as intermediate between these and the main NMC cluster. The proximity of Chagos to the NMC clusters is clearly shown at >75% similarity.

**Figure 6:**
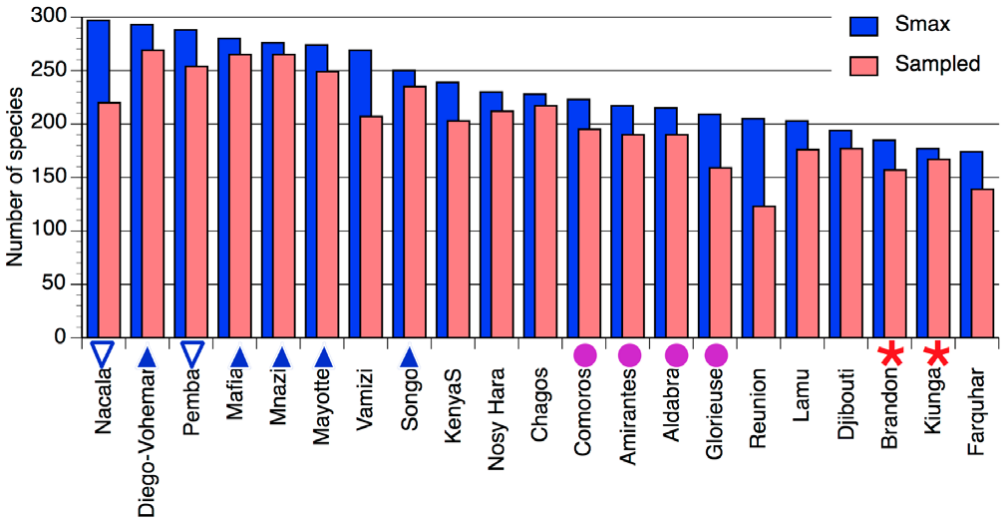
Estimated maximum species richness of reef-building corals at survey locations in the Western Indian Ocean, ordered by decreasing diversity. The graph shows Smax, derived from the Michaelis-Menten regression equation on presence/absence from a pool of 369 coral species across all locations, and the number of species sampled. Symbols against the x axis correspond to the significant cluster symbols from fig. 5.

Predicted maximum species richness (Smax) ranged from 297 (Nacala, Mozambique) to 174 (Farquhar, Seychelles) (fig. 6). The eight most species-rich locations were all grouped in the central clusters in the northern Mozambique Channel (fig. 5, NMC and NMCb), all having >250 species. The islands locations (Isl) all had similar species richness from 209–223 species. In between these two, southern Kenya, Nosy Hara (NW Madagascar) and Chagos had intermediate richness (228–239 species). Low-diversity locations ranged from Lamu and Reunion (≈205 species) to Farquhar (174 species), comprising the outliers in the cluster analysis (fig 3). Locations with the greatest differential between sampled and predicted species richness included Nacala, Vamizi, Glorieuse and Reunion (fig. 6), all four locations having the lowest sampling intensity (7–12 samples, compared to others with 15–37 samples). This is both expected by the form of the accumulation curve and an illustration of the problem of low sample size (see Methods).

### Species distributions

Of the 369 coral species identified in these surveys, the majority, just under 90%, were broadly distributed from East Africa to the West Pacific. Thirty eight species (10.3%) had Indian Ocean-only distributions (Table 3) [19], [37], [40]–[42], of which 17 are reported in the literature as Indian Ocean-wide (4.6%), 7 as restricted to the western Indian Ocean (1.9% of the total), and 14 as restricted to the north (Red Sea, Gulf of Aden, Arabian Gulf and India/Sri Lanka; 3.8%). In this survey 24 of the 38 species were broadly distributed across the WIO (6.5% of the total), 5 were only found on the mainland African coast (1.4%), 4 were found only in the Seychelles and Mascarene islands and banks (1.1%), 4 were found only in the northern Mozambique Channel (1.1%), and 1 had a disjoint distribution (0.3%). The 24 species found widespread across the WIO are listed in the literature as having more restricted western, northern or ocean-wide distributions. For example, *Acropora branch*i and *Plesiastrea devantieri* were previously reported from western and northern locations, respectively [19], but here were found throughout the WIO, and a new record now places *A. branchi* in West Sumatra [100]. Of the four corals restricted to the northern Mozambique Channel, a *Turbinaria* species with very distinct characteristics, and new to science, is most likely found in both north-west and north-east Madagascar [22], [23]. Another of these (*Acropora variolosa*) closely resembles other species in its species ‘group’ [40] (*A. austera*, *A. hemprichii* and *A. forskali*), reflecting the problems not only of field identification, but also of coral taxonomy in general (see Methods). The other two species are distinctive but very rare, each recorded in these surveys in only one or two sites in the northern Mozambique Channel, and poorly known in the literature; *Craterastrea laevis* is not listed in [19] though is now confirmed across the WIO, Chagos and Red Sea/Arabian Gulfs region [101], and *Anomastrea irregularis* lacks an underwater photograph in [19].

**Table 3:** Reported and sampled distributions of coral species restricted to the Indian Ocean.

Sampled distributions *(this study)*	Reported distributions *(literature)*				
	West	North	Ocean-wide	Subtotal	%
**Northern Mozambique Channel**	*Turbinaria* sp1 (1)		*Acropora variolosa, Anomastrea irregularis, Craterastrea laevis* (3)	4	1.1%
**Mascarenes/Seychelles**	*Ctenella chagius* (1)	*Acropora maryae, Parasimplastrea sheppardi* (2)	*Acropora lamarcki* (1)	4	1.1%
**Mainland**		*Favia albidus, Goniastrea thecata, Stylophora mammilata Porites nodifera* (4)		4	1.1%
**Disjunct**		*Porites columnaris* (1)		1	0.3%
**WIO region-wide**	*Acropora branchi, Acropora roseni, Pocillopora indiania, Stylophora madagascarensis, Horastrea indica* (5)	*Echinopora robusta, Favia lacuna, Goniastrea deformis, Plesiastrea devantieri, Porites harrisoni, Coscinaraea* zpA (6)	*Acropora appressa, Acropora forskali, Acropora hemprichi, Acropora squarrosa, Caulastrea connata, Goniastrea columella, Goniastrea peresi, Montastrea serageldini, Platygyra crosslandi, Fungia seychellensis, Gyrosmilia interrupta, Pectinia africana, Siderastrea savignyana* (13)	24	6.5%
**Subtotal**	7	13	17	37	10.0%
**%**	1.9%	3.5%	4.6%	10.0%	

Reported distributions, in columns, are derived from the literature for western, northern and ocean-wide distributions [19], [21], [40]. Sampled distributions, in rows, are as recorded in this study. The number of species in each table cell is shown in parentheses, with marginal totals, and percent of the total species number of 369.

Western Indian Ocean endemic corals are limited in number, and the majority were recorded in this study across the entire WIO. *Acropora branchi*, *A. roseni* and *Horastrea indica* are clearly established in the literature as regional endemics, while *Pocillopora indiania* is a new addition though is relatively easy to identify based on its long verrucae and tall branches [19]. *Stylophora madagascarensis* is also recently described [19], though the genus requires revision with apparently maximum genetic diversity in the WIO compared to elsewhere in the Indo-Pacific [26], [27], [43]. *Ctenella chagius* has a highly restricted distribution even within the WIO, to date known only from Chagos and Mauritius [7], [19], and in these surveys recorded in Chagos and St. Brandons Island (Mauritius).

## Discussion

### Coral species distributions

Analysis of the Red List dataset on corals identifies a number of departures from the MEOW ecoregional classification [36]. It distinguishes the West Indo-Pacific Realm from regions farther east, but draws the line west of the Andaman Seas, thus classifying the west coasts of Thailand, Malaysia, Myanmar and the Andaman and Nicobar Islands as Central Indo-Pacific in nature. The coral Red List dataset confirms the identification of the WIO as a distinct province though with significant alterations. It associates Chagos with the WIO rather than the Central Indian Ocean province, and identifies a Red Sea, Somali-Arabian Sea group (with the Persian/Arabian gulf as potentially a low-diversity/extreme environment outlier), and an India/Maldives/Sri Lanka group. Statistically, these three groups are equivalent to the MEOW provincial structure as both show equivalent levels of significance (ANOSIM R = 0.787 and R = 0.788, for 3-group and 4-group cases, p<0.001), but the three-group classification is a more realistic reflection of the similarity matrix (fig. 3b) than is the MEOW four-province classification.

The geographic scope of the field dataset is validated by the Red List analysis that joins the Chagos Archipelago to the WIO. The field dataset shows that within the WIO province the central statistically significant clusters are within the northern Mozambique Channel (NMC, figs. 3 & 4), forming a distinct core region of maximum diversity for the WIO (fig. 6). This presents a significant departure from the accepted biogeographic classifications that either separate the islands from the mainland [18], [36] with higher diversity in the islands [18], or indicate a uniform species diversity across the WIO with higher diversity in the Red Sea compared to East Africa [18], [19]. The concordance of the East African mainland and Madagascan coastlines has been noted previously [34], differentiating these from the smaller oceanic islands. This likely reflects the size of Madagascar as being more continent-like, with a coastline over 5000 km in length, but also its geological origins as a large continental fragment. Other notable features from this analysis include the similarity of the Chagos fauna to that of the WIO, the transition from the East African to Somali-Arabian fauna in northern Kenya, and the differences among the island groups in the northern Mozambique Channel, the western and northern Seychelles islands, and the Mascarene Plateau (St. Brandons, which is on the Cargados Carajos bank), southern Seychelles (Farquhar) and the Mascarene islands. These are explored in detail below, with reference to the main oceanographic features of the WIO (see Table 1).

**The center of diversity:** This study suggests a central (or ‘core’) ecoregion defined by the northern Mozambique Channel with an extension northwards to Mafia Island in Tanzania, hereafter abbreviated as the ‘NMC’. The southern boundary of the core region is not defined by this study, as the locations farthest south on the mainland coast, Nacala and Pemba at 14.5–13°S, have the highest and third highest predicted species richness (fig. 6). Similarly, only locations in the far north of Madagascar were sampled. Thus additional sampling farther south on both sides of the Mozambique Channel as well as the east coast of Madagascar is required to confirm the southern boundary. Other sources provide some insights, however. Data collected in 1999 (D. Obura, unpublished data) found lower coral species diversity at the northern end of the Primeiras Islands at Angoche, some 2° of latitude (200 km) south of Nacala. Surveys in the Primeiras and Segundas islands in 2010 (R. Salm, pers. comm.) suggest a lower diversity coral fauna, likely a result of local upwelling and freshwater and sediment influence from the Zambezi river, and the next major coral community, some 1000 km south in the Bazaruto archipelago has <200 species of hard corals [44].

The highest species richness yet reported for the WIO is in NW Madagascar in the Mitsio island group [22], just south of Nosy Hara (sampled here). In this dataset, the NE coast of Madagascar had a higher diversity than Nosy Hara on the NW coast (fig. 6), the latter potentially being lower due to poor coral habitat and the more degraded condition of the reefs there [23], [44] . Farther south though, the NW coast of Madagascar has higher habitat complexity and high connectivity due to the eddies in the NMC, so it is reasonable to expect that the sites surveyed by [22] on the NW would have equivalent or higher diversity than the NE coast site reported here. It is likely that a southern boundary for the high diversity region on the NW coast occurs by about 16°S. This latitude coincides with the narrowest part of the Mozambique Channel and the sharp decline in coral diversity on the opposite side of the channel in Mozambique. The decline in diversity may also reflect changes in the oceanography of the channel associated with this narrow constriction, which significantly influences physical processes and mesoscale dynamics of the channel [32].

Finally, on the east coast of Madagascar, well developed high-diversity coral reefs occur in the Masoala peninsula at 16°S and a decline in reef development and diversity occurs south of this point [45]. The Masoala peninsula corresponds approximately with the divide between the south-flowing East Madagascar Current and north flowing arm of the SEC that continues into the NMC [28], thus is a plausible biogeographic boundary between the high-diversity regions to the north, and lower diversity regions to the south.

**Peripheral regions:** The southern Kenya coast is shown as intermediate between the NMC locations to the south and the northern monsoon locations (Lamu and Kiunga) to the north presenting an example where coral diversity declines with latitude [19]. However in this case the latitudinal baseline is displaced to about 12°S, the latitude of the core flow of the SEC and the center of the NMC. At the southern Kenya coast, the dominance of the more diverse East African fauna appears to decline, transitioning to the less diverse Somali-Arabian fauna on the northern Kenya coast, which is classified in the Northern Monsoon Coast ecoregion [34]–[36]. Interestingly, the IUCN Red List analysis groups the Northern Monsoon Coast and central Somali coast with the WIO ecoregions (at 75% similarity, fig. 3b) and adjacent to the cluster of India/Maldives/Sri Lanka, distinguishing it strongly from the Red Sea/Gulf of Aden and other Arabian Sea ecoregions. Some of the coral species found in Kiunga and Lamu had clear affinities with the Red Sea and Gulf of Aden, and reefs in these locations are less developed due to low temperatures and high nutrients associated with upwelling in the Somali current. The reef fish fauna also shows affinities with the Gulf of Aden, with *Apolemichthys xanthotus*, the Red Sea angelfish, recorded commonly in Kiunga [46], but not farther south [47].

This analysis groups the Chagos archipelago with the core coral assemblage of the NMC (fig. 3b), and not with the more species-poor Seychelles islands nearby, nor with the Maldives and south Asian fauna as presented in other analyses [34], [36]. This is in agreement with earlier findings [18], and emphasizes the dominance of east-west flow of the SEC in the central part of the Indian Ocean, and of equatorial counter-currents which link Chagos with sites to the west while isolating it from the Maldives. Further evidence for this pattern is synthesized in [48], including independent studies on hawksbill turtles [49], the crown of thorns starfish *Acanthaster plancii* [50], two reef fish species - the peacock hind (*Cephalopholis argus* [51]) and brown surgeonfish (*Acanthurus nigrofuscus* [52]), and the coconut crab *Birgus latro* [48]. These results emphasize the role of Chagos as a stepping stone, with genetic exchange occurring in both east to west and west to east directions.

Islands within the NMC likely have a reduced species number due to area [53] and habitat complexity [54] effects. The Comorian archipelago is the product of volcanic activity, with the youngest island Grande Comore (Ngazidja) still volcanically active, and Moheli, Anjouan and Mayotte progressively older. Mayotte has a barrier reef enclosing a deep lagoon and a complex inner barrier reef system [55]. By contrast, Grande Comore, though larger, is topographically simple with coral communities growing on lava, and limited carbonate platform development on the northern and southern tips. Glorieuse, the northernmost of the French “scattered islands” (Isles Eparses) in the Mozambique Channel is a bank with two small islets. Thus coral species diversity clearly decreases along the series from Mayotte, which has comparable species richness to the continental coastlines (fig. 6) though is differentiated from them in terms of species assemblage (fig. 5), to the younger Comoro islands, and to Glorieuse.

Support for the Seychelles and Cargados/Tromelin ecoregions (see fig. 2a) is equivocal in this dataset. At the far western end of this region, the Aldabra and Amirantes groups of islands lie 150 and 900 km, respectively, from the high diversity NMC. Nevertheless, they have a very similar coral assemblage, distinct from that of the NMC. In the narrow gap between Aldabra and the Comoros, the SEC, deflected northwards by the tip of Madagascar forms a narrow jet that feeds into the EACC, in the region of the Glorioso Front [30]. This current jet may isolate the western Seychelles from the more diverse NMC and East African coast, resulting in the differentiated fauna in the western Seychelles that itself is well defined (fig. 5) and likely is consistent throughout the northern and western islands [56], [57].

St. Brandons and Farquhar islands lie in the main flow of the SEC and upstream of the NMC, though Farquhar is classified in the Seychelles ecoregion (96), and St. Brandons is in the Cargados/Tromelin ecoregion [36]. They are both shallow reef systems on broad banks with high sediment transport that is not conducive to coral growth [58]. Farquhar experiences equatorial upwelling on its southern side [59], which likely also occurs in other parts of the banks [29] and may also influence St. Brandons Island. The extensive banks of the Mascarene Plateau (the Saya de Malha, Nazareth and Cargados Carajos banks) are relatively flat with large areas between 50 and 20 m deep, and the shallowest ridges do not break the surface of the water, except at St. Brandons Island at the southern extremity. Habitat quality on these bank systems appears to be poor for reef-building corals, and those parts that have been surveyed are predominantly covered by seagrass [61]. Tromelin island, an isolated volcanic peak, was surveyed, but with only 2 sample points could not be included in this analysis. An exceptionally low diversity of 33 species was found, consistent with 26 species reported in [60], compared to an average of 107 and range of 59–155 species in 2 samples at all the other locations. This suggests its small size and isolation result in low diversity in spite of it being in the main flow of the SEC, and that it may not play an important role as a stepping stone in the region except for its limited number of (common) species. These disparate observations suggest that the depauperate coral fauna of the Mascarene Plateau (e.g. St. Brandons) and isolated islands in the Mascarene Basin (e.g. Tromelin, Farquhar) may warrant combining them in a distinct ecoregion, covering the entire Mascarene Plateau and southern Seychelles region. Further research on the oceanography and habitats of the Mascarene Plateau and Basin is needed, as existing studies on interactions between the banks and the SEC [29], [62] are suggestive of strong and highly variable physical-biological coupling that may exert control on these patterns.

To confirm the biogeographic patterns identified here requires filling a number of gaps with future surveys: along the east and west Madagascar and Mozambique coastlines to confirm the southward boundary of the core region and gradient in coral species diversity; northern Tanzania and Kenya to confirm the gradient in diversity from central Tanzania to central Kenya; the Seychelles granitic islands and Mascarene Plateau to determine subregional structure in the Seychelles region; and the Mascarene islands (Reunion, Mauritius, Rodrigues). In addition, further integration of existing datasets (e.g. [18], [42]) will enable revisions to existing large-scale analyses to test the findings of this study. Genetic research will also provide significant insights, at two levels: within species, to identify population structure across the WIO in relation to the main currents and present-day connectivity, and across species, to resolve difficulties in determining species boundaries and phylogeographic relationships that underlie present-day biogeographic patterns with historical influences. Finally, corroborating these patterns across multiple taxonomic groups will be necessary to determine the generality of these findings and fully justify the biogeographic conclusions of this study.

### The name game

The results reported here confirm the low degree of endemism in corals of the western Indian Ocean, with the added detail that endemism to subregions within the WIO is highly limited. Only seven species are apparently exclusive to the WIO (Table 3) [19, v3: p412]: five of these are found across the entire region, while 1 is limited to Chagos and the Mascarene Plateau (*Ctenella chagius*), and 1 may be limited to northern Madagascar (*Turbinaria* sp.). Of the five widespread endemics, 1 is highly distinctive phylogenetically (*Horastrea indica*), while the others are from genetically diverse and phenotypically variable Indo-West Pacific genera (*Acropora*, *Pocillopora*, *Stylophora*). The remaining 31 species occur in other parts of the Indian Ocean, but due to limited work in the WIO historically, many of these were thought to be absent from the WIO and thus endemic to these other regions. For example, *Plesiastrea devantieri* was identified recently [19], [63] and thought to be endemic to the Red Sea, but is widely distributed and common throughout the WIO. In this study 11 of these 31 species had restricted distributions within the WIO, likely a reflection of their rarity and difficulty in finding them rather than true absence, while 20 were widespread.

Comparing the results from this study with other regional analyses illustrates two of the key problems with coral species work in the last century: taxonomic confusion resulting from coral taxonomy being based on collected skeletons with until recently very little ecological, physiological or genetic foundations, and very low consistency in sampling and observations across geographic locations and observers. The scale of the first problem is underlined by the finding that most coral families and a significant number of coral genera are not monophyletic [64], [65], with a variety of sources showing the lack of correspondence between morphological and genetic data in several common genera, for example *Acropora* [66], *Platygyra* [67], *Porites* [68], *Stylophora* [27] and *Psammocora* [69], and major families, for example the Faviidae and Mussidae [70], [71]. Within the WIO, the problem is illustrated by Sheppard's [18] reduction of a compiled list of 796 nominal species names from the literature to 439 synonymzed names that he judged to be a correct reflection of species taxonomy. The natural history method applied in this study, of iterative reanalysis of in situ species and ecomorph identifications across reef habitats and locations across a consistent biogeographic region, referenced by a ‘voucher’ collection of underwater photographs [72], has the benefit of a more holistic inclusion of potential variation in a species appearance under many different settings. That these identifications are not referenced to collected voucher specimens is a major weakness in classical taxonomic terms, though the problems of this latter approach demand exploration of alternative approaches (see Methods).

**Figure 7:**
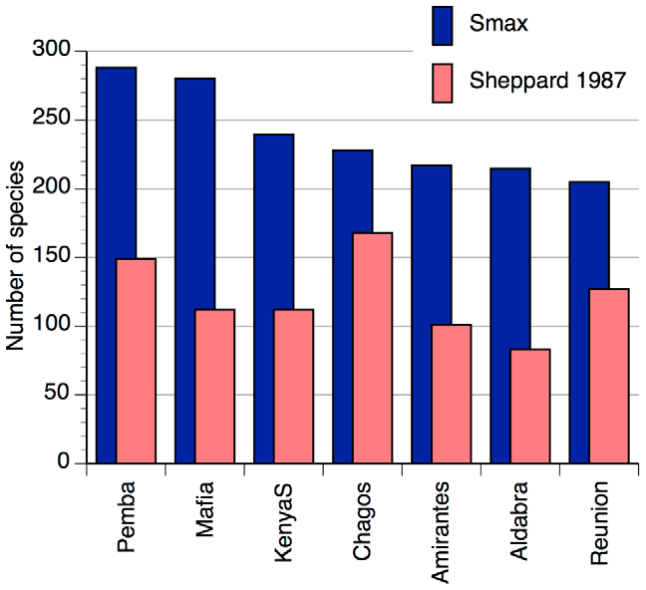
Comparison of coral species richness for areas in the Western Indian Ocean, between this study and Sheppard [**18**]. Reported species richness is from [18] and estimated asymptotic number of species (Smax) is from this study. Maximum Smax is shown where there were multiple locations from this study corresponding to areas in [18].

The second problem, of low consistency in sampling and observations among different studies and across geographic locations is illustrated by comparing areas included in this study with [18]. While Sheppard compiled 439 valid coral species names, this study recorded 413 species, of which 369 were named with stable identifications. By contrast, the site-level richness reported in this study was from 1.5 to 3 times higher than those that Sheppard had access to (fig. 7), with the highest correspondence being for the Chagos archipelago, and lowest for mainland sites in Tanzania and Kenya. Sources for Sheppard's numbers varied considerably, from a single PhD thesis from the mid-1970s for northern Tanzania [1] to multiple higher level studies for the Red Sea [73]–[75], and Sheppard's own consistent body of work for Chagos [76].

This comparison also introduces the question of what the total pool of coral species may be for the area covered by this study. Veron and Turak recorded a total of 323 species from their study, which combined with past studies gave a total richness of 380 for Madagascar [22]. This study compiled 369 identified species across the whole WIO region, with an additional 44 species that were un-named, for a total of 413 species. The IUCN Red List has a total of 398 species for the WIO [37]. The difficulties of species identification in several genera also suggests that further taxonomic work in the WIO will add several tens of names to existing species lists. Further, additional detailed sampling particularly in the NMC will likely add more species, as has been done in 2011 for *Acropora* specimens from Mayotte [77]. Therefore, it is reasonable to expect that the regional species pool for the WIO will climb higher, and may approach 450 species, equivalent in diversity to areas such as the Andaman Sea, Coral Sea and northern Great Barrier Reef.

### Drivers of regional patterns

Several patterns can be distinguished, integrating oceanographic drivers with the diversity patterns presented in Table 1. The net result is that genetic material may be transported into the WIO on the SEC and retained within the northern Mozambique Channel by eddies within the channel, resulting in accumulation of genetic and species diversity, equivalent to mechanisms identified that maintain high diversity in the Coral Triangle [19], [20]. Export of this material north and south occurs along essentially linear transport corridors, resulting in attenuation of diversity outside of the core region and transitions to lower-diversity colder-water systems to the north and south. Some circular flow is possible in gyres and return flows north and south of the SEC in the Seychelles and Mascarene islands. Some interaction with the northwestern regions of the Indian Ocean occurs as a result of monsoon oscillations in currents. The net result of these is a ‘core region’ of high diversity within the NMC, set in a coherent biogeographic province at the level of the WIO that includes the Chagos archipelago.

These drivers of connectivity and the species patterns presented here suggest revisions to existing ecoregional classifications should be considered. Inconsistencies among various biogeographic classifications of the WIO based on different factors have been noted [34], such as between those based on general climate [78] versus hydrography [79]. However, no further analytical work was carried out on these issues, and these inconsistencies have been carried forward into practical ecoregional exercises focused on conservation planning, splitting the core ecoregion of the WIO down the middle of the Mozambique Channel [35], [36]. Further, a focus on carbon and highly productive coastal systems that have defined the Large Marine Ecosystem (LME) approach [80] has guided global marine ecosystem planning efforts. This has resulted in regional structures that in some cases are inconsistent with tropical oligotrophic systems; in the WIO, the Agulhas and Somali Current LMEs extend outside of the regions in which these currents have a sphere of influence, in an upstream direction, and paradoxically split the WIO at its primary unifying feature, the South Equatorial Current. To reconcile these inconsistencies revisions to province and ecoregion level boundaries are proposed (Table 4, fig. 2b). In considering these further, other species groups and biogeographic processes need to be evaluated, to improve understanding of diversity patterns in the Indian Ocean and catalyze new work to fill in the glaring gaps in knowledge about the marine biodiversity of the region [81], [82].

**Table 4:** Proposed changes to province, ecoregion and Large Marine Ecosystem classifications in the Indo-West Pacific Realm, based on reef-building corals.

**Realm-level change**		
Andaman Seas	The Andaman Seas province should be placed in the Central Indo-Pacific Realm as its fauna is most closely related to the Sunda/Java/South China Sea region, the broader West Pacific and even Polynesia than it is to adjacent locations in the Central Indian Ocean, Bengal Bay and South India.	Primary (RL)
**Province-level changes**		
Chagos	Chagos should be grouped with the WIO province, emphasizing the zonal oceanographic influence, its role as a stepping stone in both easterly and westerly directions, and that this pattern holds across multiple taxonomic groups.	Primary (field & RL)
Somali, Arabian Sea and Red Sea	The Northern Monsoon coast and central Somali coasts should both be grouped with the WIO province. The clusters (fig. 3b) for the Persian, Arabian, Oman, Gulf of Aden and Red Sea ecoregions are geographically disjoint, suggesting that together they might form a single province.	Primary (RL)
Maldives, India & Sri Lanka	The Maldives, south and west India and Sri Lanka may be grouped in a single province.	Primary (RL)
**Ecoregion-level changes**		
Northern Mozambique Channel (NMC)	The basic structure of the marine ecoregions of the WIO should be altered from the paradigm that separates island and mainland systems, to one that has the Northern Mozambique Channel (NMC) as a core region, likely with a concentric pattern of other ecoregions reflecting flow and dispersal out of the NMC. The East African Coral Coast (95) and North and West Madagascar (100) must be split to accommodate joining of their parts that fall in the NMC core region identified by this study. For the former this includes from Mafia Island (Tanzania) to Nacala (Mozambique), and for the latter, the NW and upper NE coasts of Madagascar, plus the Comoros archipelago, Glorieuse island and nearby banks (Zelee and Geyser).	Primary (field & RL)
East African Coast	The East African Coral Coast ecoregion (95) must be split to accommodate the NMC core ecoregion, resulting in a northern one including northern Tanzania and southern Kenya, and a southern one from the Primeiras and Segundas Islands to Delagoa in Mozambique. Sampling limitations of this study require further work to elucidate these.	Primary (field) (Kenya/Tanzania); Secondary (Mozambique )
West Madagascar coast and southern Mozambique Channel	Similarly, the North and West Madagascar ecoregion (100) must be split, leaving the central-west coast of Madagascar from approximately 16°S at the narrowest part of the Mozambique Channel to Tulear in the south. It is also unclear if a single ecoregion may span the southern Mozambique Channel to Mozambique (combined with the Primeiras-Segundas to Delagoa region, above), or if these may be two separate ecoregions on the east and west of the channel.	Secondary [36]
East and South Madagascar	Recent work suggests the extreme south of Madagascar is temperate in nature, and should be a distinct ecoregion, different from both the East and Southwest costs of Madagascar.	Secondary [99]
Seychelles and the banks on the Mascarene plateau	Low diversity at Farquhar suggests it and the isolated, low habitat/area south-central islands (e.g. Providence, Agalega) may be more appropriately placed in the banks ecoregion (Cargados/Tromelin, 97). Further work on the geology, oceanography and biodiversity of the Mascarene Basin and Plateau, will be needed to address this question.	Primary (field)/Secondary [29], [58], [59], [60], [62]
**Large Marine Ecosystems (LME)**		
Agulhas-Somali Current LME)	The boundaries of the WIO province in the north and south are coherent with the primary features of the named LME systems - the Somali and Agulhas currents, respectively. But these have limited influence on the core zone of the WIO as they are downstream of it. This suggests revisions to the LMEs of the region may be warranted, to distinguish an LME considering interactions of the South Equatorial Current with the Mascarene Plateau and the mesoscale dynamics of the Mozambique Channel.	Primary (field)/Secondary [29]–[32], [80]

Province and ecoregion names and numbers follow the MEOW classification [36] (see caption to fig. 2), while the suggested changes are illustrated in fig. 2b. Findings are labeled by whether they are primary (supported by data from the field or the IUCN Red List (RL), along with statistical analysis) or secondary, inferred from primary findings of this study and listed sources. Both levels of findings pose hypotheses for further research and confirmation.

This analysis has focused exclusively on present-day oceanography as the driving force in establishing the biogeographic patterns in the WIO. Historical forces are also important [83], [84], though beyond the scope of this analysis, and likely have a significant influence on the diversity patterns and phylogenetic origins of species of the western Indian Ocean [85].

## Methods

Global coral species distributions were compiled for the IUCN Red List of Threatened Species [37] as described in [38], and extracted as presence/absence records by ecoregion [36]. Field distributions of coral species were obtained for 22 locations in and adjacent to the WIO, from October 2002 to September 2011 (fig. 2a, Table 2), though one location, Tromelin, was excluded from analyses due to insufficient sample size. Due to variation in the source of funding and principal objectives of each survey, differing numbers and duration of in-water surveys for coral species were possible. The intensity of sampling focused on coral species presence/absence varied from entire dives dedicated to this single task and lasting 45 to 70 minutes, to multi-tasking dives; of the latter, only those dives in which at least 15 minutes of the dive were dedicated to species observations are included in this analysis. During dedicated species observation dives, all coral species encountered were recorded, though with an emphasis on searching for new species not recorded on prior dives in the same expedition. On multi-tasking dives, only new species not previously observed were recorded. The overall goal of these surveys was to build up a total list of coral species across all dives of a survey trip and across multiple habitats. Surveys targeted shoreline, lagoon and fore-reef habitats, the dominant habitats on reefs in the WIO. Minor habitats, such as in high-sediment environments near estuaries, or isolated pinnacles were generally not sampled due to logistical limitations.

Coral species were identified *in situ*, using digital photography to record voucher photographs of species [72], to assist with species identification using resources based on colour underwater photographs (e.g. [19], [40]). For genera in which a secondary reference appeared more consistent with regional patterns and colony appearance, these were used, e.g. for Red Sea corals [9] and the genus *Psammocora* [69]. The method used here, focusing on “ecomorph/biogeographic observation” emphasizes a natural historical approach. This can be contrasted with three alternative approaches that can be labeled as macromorphogical (the basis of scleractinian taxonomy until recently [19], [86], [87]), micromorphological [26], [70], [88], [89] and genetic [26], [27], [64]–[71]. Each of these approaches has its strengths and weakness, and history of use, and can be matched with specific contexts of objectives and resources. Recent findings by several authors that “*most macromorphologic characters that have traditionally been used to distinguish scleractinian families, genera, and species … are only of limited use in scleractinian phylogeny reconstruction and classification*” (paraphrased from [88], and see [64]) made using the classical taxonomic approach in this study unwise, as well as it being impractical due to the budgets available, with its requirements of collection, archiving, regional facilities and expertise to work on the specimens. The genetic and micro-morphological approaches have matured over the period of this study, and are prohibitive financially and technically for application across the breadth of the material and geography of this study. Thus the approach used here, informed by incremental findings from researchers applying the other approaches within specific taxonomic groups and/or geographies (e.g. [66]–[71]), best matched the opportunities and constraints of this study. It is clear that future work on coral phylogeny and systematics will require greater synthesis across these approaches [90]. Ethical clearance for in-water observation of benthic invertebrates was obtained from the Kenya National Council for Science and Technology.

### Analysis

Analysis of species distributions and accumulation curves were conducted using Primer v 6.0 [39], [91]. Presence/absence data were used to generate similarity matrices using the Bray-Curtis similarity index between a) provinces and ecoregions for IUCN Red List data, and b) sample locations from field surveys (Table 2, Dataset S1). The Bray-Curtis index was selected as it is among the most widely used and researched indices [92] and because other indices commonly used for presence/absence data are either presence/absence forms of this index (e.g. Sorensen's index), or gave very similar results when trialed on this dataset (Jaccard's index). The resulting similarity matrices were analyzed by cluster analysis (group averaging method) and multi-dimensional scaling (MDS), and the significance of the similarity matrices assessed using ANOSIM (Analysis of Similarities).

Species accumulation curves were derived from the same presence/absence field data. For locations with surveys from multiple years, all dive samples were used as independent samples, and for all locations the order of samples was randomized and resampled 999 times (Primer v 6.0). A variety of estimation procedures were investigated with this dataset, to select the one least impacted by variation in sampling and other factors. Sensitivity of maximum diversity (richness) estimators is a large field, as estimators have varying assumptions and are based on different aspects of diversity, rarity or presence [93]–[95]. In general, all estimators improve with increased sampling, and in most cases estimated maximum diversity initially increases with sample size. On theoretical considerations, Smax from the Michaelis-Menten equation fit this dataset best, as it is independent of the rarity of a species, being based solely on presence. On empirical considerations it was also found to be the least biased by the number of samples and the most stable statistic to use for estimated maximum richness of locations (Table 5). The second parameter of the Michaelis-Menten equation, B, is the level of sampling at which half of Smax is reached. This varied from 2–7 for all locations in this dataset (Table 2); for locations in which species sampling was the exclusive focus, B varied from 2–3. Thus B appears to reflect variability in method and sampling differences between surveys, and how these (among other factors) affect the initial slope of the curve (how quickly species observations are accumulated), but with less effect on the estimate of maximum diversity.

**Table 5:** Maximum diversity (species richness) estimators applied to the NE Madagascar surveys, based on random samples of 6, 7, 8, 10, 12, 15, 20, 25, 30 and 36 samples*.

Samp	Sobs	Chao		Jacknife		Boot-	UGE	MM	Smax
les		1	2	1	2	strap			
6	212	269.6	269.6	267.8	291.7	238.7	212	211.8	286.3
7	210	249.6	249.6	262.3	277.9	236.0	210	210.2	286.4
8	221	253.7	253.7	267.4	279.5	244.6	221	221.2	282.5
10	235	291.7	291.7	291.7	317.2	261.9	235	231.3	285.9
12	236	264.9	264.9	279.1	289.9	257.8	236	234.0	279.1
15	242	265.1	265.1	282.1	289.4	263.2	242	243.8	298.3
20	252	281.4	281.4	295.7	306.4	274.3	252	248.8	290.6
25	257	269.9	269.9	285.3	291.4	273.0	257	255.9	287.6
30	262	274.0	274.0	285.7	290.8	275.5	262	263.0	292.9
36	269	285.9	285.9	294.3	300.5	282.6	269	268.0	292.7
max	269.0	291.7	291.7	295.7	317.2	282.6	269.0	268.0	298.3
m	239.6	270.6	270.6	281.1	293.5	260.8	239.6	238.8	288.2
sd	20.7	13.3	13.3	11.8	11.8	16.3	20.7	20.5	5.6
CV	0.086	0.049	0.049	0.042	0.040	0.063	0.086	0.086	0.019
range	59.0	42.1	42.1	33.4	39.3	46.6	59.0	57.8	19.2

*Sobs* - Curve of observed species counts; *Chao 1* - Chao's estimator based on number of rare species; *Chao 2* - Chao's estimator using just presence-absence data; *Jacknife 1* - Jacknife estimator based on species that only occur in one sample; *Jacknife 2* - Second order jacknife estimator; *Bootstrap* - Bootstrap estimator based on proportion of quadrats containing each species; *UGE* - Calculated species accumulation curve; *Michaelis-Menten* - MM are points on the curve fitted to Sobs, with curve parameter Smax = asymptote at infinity [39], [88]–[92].

Notes:

* The only condition for including a sample in these subsamples was that for cases of <15 samples, only full-diversity samples were selected by excluding minor samples in which <40 coral species were recorded overall (see methods for cases where sampling of species was constrained by other objectives during a dive, so records were taken only of new species seen in that dive).
